# A model for spatial variations in life expectancy; mortality in Chinese regions in 2000

**DOI:** 10.1186/1476-072X-6-16

**Published:** 2007-05-02

**Authors:** Peter Congdon

**Affiliations:** 1 Department of Geography, Queen Mary, University of London, Mile End Rd, London E1 4NS, UK

## Abstract

**Background:**

Life expectancy in China has been improving markedly but health gains have been uneven and there is inequality in survival chances between regions and in rural as against urban areas. This paper applies a statistical modelling approach to mortality data collected in conjunction with the 2000 Census to formally assess spatial mortality contrasts in China. The modelling approach provides interpretable summary parameters (e.g. the relative mortality risk in rural as against urban areas) and is more parsimonious in terms of parameters than the conventional life table model.

**Results:**

Predictive fit is assessed both globally and at the level of individual five year age groups. A proportional model (age and area effects independent) has a worse fit than one allowing age-area interactions following a bilinear form. The best fit is obtained by allowing for child and oldest age mortality rates to vary spatially.

**Conclusion:**

There is evidence that age (21 age groups) and area (31 Chinese administrative divisions) are not proportional (i.e. independent) mortality risk factors. In fact, spatial contrasts are greatest at young ages. There is a pronounced rural survival disadvantage, and large differences in life expectancy between provinces.

## Background

This paper develops a model for mortality contrasts between 31 administrative divisions of China. Despite major gains in life expectancy in China and improved health and living standards, there is evidence of considerable social and health inequality [1], and poor access to health care in rural and less developed areas [2]. As a result, health and life expectancy improvements have been uneven, typically greater in developed eastern and southern areas of China.

Assessment of age-related inequalities in mortality is therefore important, and life table analysis is often used to this end. However, conventional life table analysis proceeds by independent analysis of each region, often by spreadsheet; for example, see the Population Analysis Spreadsheets developed by the US Census Bureau [3]. This approach to life table analysis takes no account of spatial structure in mortality risks due to socio-economic and environmental factors that affect neighbouring regions similarly. Life table analysis most commonly also adopts (albeit often implicitly) a saturated fixed effects model that does not model correlation in mortality rates between adjacent age groups, and is heavily parameterised.

Here the modelling approach pools strength over ages and regions using random effects methods that recognize correlations between adjacent ages and areas. The number of parameters involved in the model becomes an unknown, often referred to as an effective parameter total, and with a suitably designed model this total may be considerably less than the parameters needed under fixed effects approaches [4].

On the other hand, it is possible that a mortality model oversimplifies such that predictions from it do not accurately reproduce the observed mortality data. Many models in the disease mapping literature assume multiplicative age and area effects, under which area effects on mortality relative risk are constant across age groups [5,6]. The modelling approach here, while relatively parsimonious, explicitly recognizes that age and area effects in observed mortality schedules may not be multiplicative (or additive in the logs of mortality risk), in which case predictive discrepancies will be apparent when a multiplicative model is used. Instead an interaction scheme between age and area dimensions is proposed by adapting the bilinear interaction scheme of Lee and Carter [7], who consider mortality forecasts in age and time dimensions.

### Data sources

As mentioned by Banister & Hill [8] the most complete mortality data for China to date have been gathered from all households in the three most recent nationwide censuses of 1982, 1990, and 2000. Over a yearly period ending at the census date, information about household deaths was collected by single year of age and sex. These are population wide collections of death details parallel to the full population enumeration provided by the census. Here the focus is on mortality data over a calendar year (1.11.1999 to 31.10.2000) collected in conjunction with the 5^*th *^population census of China conducted on November 1, 2000. The mortality data can be disaggregated to a relatively low geographic level [9], but here the framework is the 31 administrative divisions of China. These divisions are a mix of 22 provinces, 5 autonomous regions and 4 direct-controlled municipalities (Beijing, Tianjin, Shanghai, and Chongqing), though with status equal to that of the provinces [10]. Taiwan is not included. Specific data sources are the Complete Collection of Provincial Population Census Data Assemblies (2000 Census), available from the University of Michigan China Data Center, and in particular two sets of tables:

Tables 1-7a, 1-7b and 1-7c, populations by age, sex and for cities/towns/rural within each division;

Tables 6-1a, 6-1b, 6-1c, deaths over 1.11.1999 to 31.10.2000 by age, sex and for cities/towns/rural within each division.

Populations and deaths in towns and cities within each of the 31 divisions are amalgamated to provide a simplified comparison of urban and rural mortality.

While the deaths data collected in conjunction with recent China censuses are the most complete available, there is still under-recording. A full discussion of death under-counting is provided by Bannister & Hill [8] who obtained correction factors using the general growth balance method [11]. In deriving mortality rates and life expectancies in this paper, adjustment factors from the paper by Banister and Hill are applied to correct for mortality under-recording. Specifically, populations at risk are reduced to correspond to recorded deaths; thus the male mortality adjustment factor was 1.113, so recorded deaths were retained as the response variables but analyzed in relation to census populations scaled by 1/1.113 = 0.898; for females the adjustment factor is 1.181 so female populations are scaled by 0.847. As reported below (Table 5) China wide life table estimates from the models used here are close to those from Banister and Hill that correct for death undercount.

So the observed data consists of deaths and (scaled) populations differentiated by age (21 groups from 0–4,5–9 through to 100+), by gender, and by an urban-rural categorisation of populations within each administrative division. For simplicity we refer to the administrative divisions simply as divisions below. To provide some background on the varying socio-economic character of these divisions, Appendix 1 tabulates comparative data on indices of literacy, rurality, income and employment. The wide contrasts in development and living standards are relevant to interpreting mortality differences.

Other analyses of the 2000 census mortality data are provided by Heilig [12]. Lai [13], and Cai [9], while Lai et al [14] provide analysis of corresponding data for 1990. These analyses adopt conventional fixed effects approaches, in contrast to the comprehensive approach to smoothing over areas and ages as obtained by the random effects methods used in this paper. Whereas a fixed effects model of conventional life table analysis would involve 31 × 21 × 2 × 2 = 2604 parameters when applied to the data in this paper, the approach developed here is shown to be considerably more parsimonious, while also reproducing the data accurately.

A Bayesian approach [15] is adopted which fully allows for parameter uncertainty.

With repeated sampling from the posterior density of parameters using Monte Carlo Markov Chain (MCMC) methods, the stochastic variation in life table parameters to be readily obtained: for example, one may obtain 95% intervals for division life expectancies or more specific outputs such as the difference in urban as against rural life expectancy within each division.

## The life table model

Let i denote division, x denote age, s denote gender (1 = females, 2 = males) and r denote urban vs rural populations in each division (r = 1 for urban, 2 for rural). The data are deaths y_*risx *_and scaled populations P_*risx*_. Because of the aggregated spatial scale and relatively large death counts involved, the life table model assumes deaths y_*risx *_to follow a negative binomial density, namely

p(yrisx|ξrisx,α)=Γ(α+yrisx)Γ(α)Γ(yrisx+1)(αα+ξrisx)α(ξrisxα+ξrisx)yrisx
 MathType@MTEF@5@5@+=feaafiart1ev1aaatCvAUfKttLearuWrP9MDH5MBPbIqV92AaeXatLxBI9gBaebbnrfifHhDYfgasaacH8akY=wiFfYdH8Gipec8Eeeu0xXdbba9frFj0=OqFfea0dXdd9vqai=hGuQ8kuc9pgc9s8qqaq=dirpe0xb9q8qiLsFr0=vr0=vr0dc8meaabaqaciaacaGaaeqabaqabeGadaaakeaacqWGWbaCcqGGOaakcqWG5bqEdaWgaaWcbaGaemOCaiNaemyAaKMaem4CamNaemiEaGhabeaakiabcYha8HGaciab=57a4naaBaaaleaacqWGYbGCcqWGPbqAcqWGZbWCcqWG4baEaeqaaOGaeiilaWIae8xSdeMaeiykaKIaeyypa0ZaaSaaaeaacqqHtoWrcqGGOaakcqWFXoqycqGHRaWkcqWG5bqEdaWgaaWcbaGaemOCaiNaemyAaKMaem4CamNaemiEaGhabeaakiabcMcaPaqaaiabfo5ahjabcIcaOiab=f7aHjabcMcaPiabfo5ahjabcIcaOiabdMha5naaBaaaleaacqWGYbGCcqWGPbqAcqWGZbWCcqWG4baEaeqaaOGaey4kaSIaeGymaeJaeiykaKcaamaabmaabaWaaSaaaeaacqWFXoqyaeaacqWFXoqycqGHRaWkiiaacqGF+oaEdaWgaaWcbaGaemOCaiNaemyAaKMaem4CamNaemiEaGhabeaaaaaakiaawIcacaGLPaaadaahaaWcbeqaaiab=f7aHbaakmaabmaabaWaaSaaaeaacqWF+oaEdaWgaaWcbaGaemOCaiNaemyAaKMaem4CamNaemiEaGhabeaaaOqaaiab=f7aHjabgUcaRiab=57a4naaBaaaleaacqWGYbGCcqWGPbqAcqWGZbWCcqWG4baEaeqaaaaaaOGaayjkaiaawMcaamaaCaaaleqabaGaemyEaK3aaSbaaWqaaiabdkhaYjabdMgaPjabdohaZjabdIha4bqabaaaaaaa@8BEF@

where *ξ*_*risx *_represents the modelled mortality count. The negative binomial is obtained as the marginal density under an overdispersed Poisson model namely *y*_*risx *_~ *Po*(*μ*_*risx*_), with means *μ*_*risx *_themselves following a gamma distribution. Specifically *μ*_*risx *_~ *G*(*α,α/ξ*_*risx*_), whereby *E*(*μ*_*risx*_) = *ξ*_*risx*_, *Var*(*μ*_*risx*_) = ξrisx2
 MathType@MTEF@5@5@+=feaafiart1ev1aaatCvAUfKttLearuWrP9MDH5MBPbIqV92AaeXatLxBI9gBaebbnrfifHhDYfgasaacH8akY=wiFfYdH8Gipec8Eeeu0xXdbba9frFj0=OqFfea0dXdd9vqai=hGuQ8kuc9pgc9s8qqaq=dirpe0xb9q8qiLsFr0=vr0=vr0dc8meaabaqaciaacaGaaeqabaqabeGadaaakeaaiiGacqWF+oaEdaqhaaWcbaGaemOCaiNaemyAaKMaem4CamNaemiEaGhabaGaeGOmaidaaaaa@3545@/*α *and

*Var*(*y*_*risx*_) = *E*[*Var*(*y*_*risx*_|*μ*_*risx*_)] + *Var*[*E*(*y*_*risx *_|*μ*_*risx*_)] = *ξ*_*risx *_+ ξrisx2
 MathType@MTEF@5@5@+=feaafiart1ev1aaatCvAUfKttLearuWrP9MDH5MBPbIqV92AaeXatLxBI9gBaebbnrfifHhDYfgasaacH8akY=wiFfYdH8Gipec8Eeeu0xXdbba9frFj0=OqFfea0dXdd9vqai=hGuQ8kuc9pgc9s8qqaq=dirpe0xb9q8qiLsFr0=vr0=vr0dc8meaabaqaciaacaGaaeqabaqabeGadaaakeaaiiGacqWF+oaEdaqhaaWcbaGaemOCaiNaemyAaKMaem4CamNaemiEaGhabaGaeGOmaidaaaaa@3545@/*α*

The initial multiplicative model 1 for mortality counts *ξ*_*risx *_involves

a) an overall mortality level parameter by gender s, *κ*_*s*_;

b) parameters *η*_*xs *_to represent the age-sex mortality rates for age × and gender s; these are assumed to follow a first order random walk that reflects correlation in rates between successive ages;

c) parameters *γ*_*s *_to represent a gender specific rural mortality differential;

d) division effects *φ*_*is *_to represent spatially correlated mortality contrasts that are likely to be gender differentiated.

The age parameters *η*_*xs *_describe the typical age profile of mortality: relatively high child mortality, followed by low mortality for older children and young adults, and then rising at older ages. The age parameters in model 1 apply across all provinces in line with the multiplicative model – the validity of which the analysis here is seeking to assess. For example, it may well be that some degree of regional variation in age effects is in fact present in the Chinese mortality data, and model elaborations explained below allow for this. The final set of parameters *φ*_*is *_reflect unmeasured risk factors for (or influences on) mortality that are themselves spatially patterned [16]. For example, differences in environment, health care, climate, economic development and so on are likely to be spatially correlated.

Then model 1 specifies

log(*ξ*_1*isx*_) = log(*P*_1*isx*_) + *κ*_*s *_+ *η*_*xs *_+ *φ*_*is *_

log(*ξ*_2*isx*_) = log(*P*_2*isx*_) + *κ*_*s *_+ *γ*_*s *_+ *η*_*xs *_+ *φ*_*is *_

where P_*risx*_are scaled populations from the 2000 China Census. Detailed assumptions about the age and spatial effects (in terms of prior densities) are considered in Appendix 2.

In model 1 the age effects *η*_*xs *_are assumed to be independent of area (division) effects, in line with the widely applied multiplicative model [5]. Multiplicativity refers to the original mortality risk scale, when all effects are exponentiated in (3). The multiplicative assumption leads to a parsimonious model but the actual mortality pattern may not conform to the simplifying assumption of a uniform age gradient through all divisions. For example, it may be that age related discrepancies from the multiplicative model occur, in line with the overall mortality level in a division. So under a multiplicative model, infant and child mortality may be underpredicted in relatively backward high mortality divisions and overpredicted in the more developed lower mortality divisions.

To reflect such possibilities a multiplicative interaction between area and age is introduced in the log relative risk scale. This provides a more general though still relatively parsimonious model (model 2), namely

*log*(*ξ*_1*isx*_) = *log*(*P*_1*isx*_) + *k*_*s *_+ *η*_*xs *_+ *φ*_*xs*_*θ*_*is *_

*log*(*ξ*_2*isx*_) = log(*P*_2*isx*_) + *k*_*s *_+ *γ*_*s *_+ *η*_*xs *_+ *φ*_*xs*_*θ*_*is *_

where the *θ*_*is *_represent area mortality contrasts, especially for particular age groups. In fact, for parameter identification the *θ*_*is *_sum to zero, while the *φ*_*xs *_sum to 1, namely ∑xϕx1=∑xϕx2=1
 MathType@MTEF@5@5@+=feaafiart1ev1aaatCvAUfKttLearuWrP9MDH5MBPbIqV92AaeXatLxBI9gBaebbnrfifHhDYfgasaacH8akY=wiFfYdH8Gipec8Eeeu0xXdbba9frFj0=OqFfea0dXdd9vqai=hGuQ8kuc9pgc9s8qqaq=dirpe0xb9q8qiLsFr0=vr0=vr0dc8meaabaqaciaacaGaaeqabaqabeGadaaakeaadaaeqbqaaGGaciab=v9aQnaaBaaaleaacqWG4baEcqaIXaqmaeqaaaqaaiabdIha4bqab0GaeyyeIuoakiabg2da9maaqafabaGae8x1dO2aaSbaaSqaaiabdIha4jabikdaYaqabaaabaGaemiEaGhabeqdcqGHris5aOGaeyypa0JaeGymaedaaa@3FA4@. Higher weights for a particular age express the fact that these ages are particularly subject to the spatial variation expressed in *θ*_*is*_. For example, divisions with high *θ*_*is *_may tend to have high child or young adult mortality (this anticipates findings later in the paper), and so the age-sex *φ*_*xs *_parameters will be higher at such ages.

An analogous scheme, but in an age-time rather than age-area context, is the Lee-Carter model used for mortality forecasting [7]. For death counts y_*xt *_with negative binomial or Poisson means *ξ*_*xt *_the Lee-Carter model would involve a log-bilinear form

log(*ξ*_*xt*_) = *k *+ *η*_*x *_+ *φ*_*x*_*θ*_*t*_

where age weights *φ*_*x *_are highest for those age groups showing most improvement according to mortality trend parameters *θ*_*t *_which are typically correlated in time.

The overall parameter combination *φ*_*xs*_*θ*_*is *_in equation (4) provides a relatively parsimonious representation of age-sex-province mortality effects involving 104 parameters, that avoids using 31*21*2 = 1302 age-area interaction parameters *ψ*_*xis*_. If model discrepancies remain despite the generalisation in equation 4 (e.g. in predictions matching observations), one might introduce *ψ*_*xis*_, but this is likely to be at the cost of model parsimony if applied across all ages. The option considered here is to selectively add area effects *ψ*_*xis *_for age bands x according to an age level binary indicator *δ*_*x*_.

So with a low prior probability *π*_*δ *_(e.g. *π*_*δ *_= 0.05) that *δ*_*x *_= 1, additional area-gender effects specific to the selected age are added. This might be expected to occur particularly for any ages where the mechanism in model 2 still leaves discrepancies in fit. The potential additional effects *ψ*_*xis *_are assumed to be normally distributed random effects with mean zero and an age specific variance parameter *ζ*_*x*_, namely *ψ*_*xis *_~ *N*(0, *ζ*_*x*_). So for model 3, one has *δ*_*x *_~ *Bern*(*π*_*δ*_) and

*log*(*ξ*_1*isx*_) = log(*P*_1*isX*_) + *κ*_*s *_+ *η*_*xs *_+ *φ*_*xs*_*θ*_*is *_+ *δ*_*x*_*ψ*_*xis *_

*log*(*ξ*_2*isx*_) = log(*P*_2*isX*_) + *κ*_*s *_+ *γ*_*s *_+ *η*_*xs *_+ *φ*_*xs*_*θ*_*is *_+ *δ*_*x*_*ψ*_*xis *_.

## Findings

Models are estimated using the WINBUGS program [17]. Methods of assessing model fit and adequacy are discussed in Appendix 3, and model fit is summarised in Table 1. For all models, two chains were run for 5000 iterations with convergence by 1000 using Gelman-Rubin scale reduction factors [18]. The Deviance Information Criterion (DIC) – see Appendix 3-under the multiplicative model 1 is 35860 with 99 estimated parameters, while the log of the pseudo marginal likelihood is -17934 (Table 1). Just over 5% of the cases are not included in the 95% intervals of predictions *y*_*rep*,*risx*_. So in overall terms the model is producing predictions that are concordant with the data. On the other hand, an examination of the pattern of model discrepancies at the age group level (Table 2) shows that a relatively high number of death counts for ages 0–4 and the oldest ages are not predicted satisfactorily; note that there are 2604/21 = 124 observations in each age group so under model 1, 38 of the 124 death counts in age group are not satisfactorily predicted.

**Table 1:** Model Fit Summary

	Pseudo Marginal Likelihood	Mean Deviance	Estimated Parameters	DIC	Number (and %) of observations not contained within 95% intervals of y_rep,risx_	
					No.	%

Model 1	-17934	35761	99	35860	138	5.3
Model 2	-17398	34656	82	34738	132	5.1
Model 3	-17227	33900	346	34246	89	3.4

**Table 2:** Predictive Match by Age Band

	Total observations not within 95% intervals of y_rep,risx_			Average of log(CPO)		
Age band	Model 1	Model 2	Model 3	Model 1	Model 2	Model 3

0–4	38	30	13	-9.0	-8.3	-8.3
5–9	6	3	0	-5.6	-5.2	-5.2
10–14	4	0	0	-5.4	-5.1	-5.0
15–19	11	12	17	-6.0	-5.9	-6.0
20–24	12	6	10	-6.3	-6.1	-6.2
25–29	3	2	6	-6.5	-6.3	-6.3
30–34	1	1	4	-6.5	-6.3	-6.3
35–39	1	2	2	-6.5	-6.3	-6.3
40–44	1	1	1	-6.6	-6.4	-6.3
45–49	0	1	1	-6.9	-6.8	-6.7
50–54	0	0	1	-7.1	-7.0	-6.9
55–59	0	1	3	-7.3	-7.2	-7.2
60–64	0	2	3	-7.8	-7.7	-7.6
65–69	1	3	4	-8.1	-8.0	-8.0
70–74	1	1	6	-8.3	-8.2	-8.2
75–79	1	2	2	-8.3	-8.1	-8.0
80–84	2	2	6	-8.1	-7.9	-7.9
85–89	6	5	2	-7.7	-7.4	-7.3
90–94	7	8	4	-6.8	-6.5	-6.4
95–99	20	23	0	-5.8	-5.6	-5.2
100+	23	27	4	-4.1	-4.0	-3.8
All*	138	132	89	-6.9	-6.7	-6.6

* Gives total observations (in 2604) not within 95% intervals of y_rep _and average log(CPO) over all 2604 cases

Application of model 2, as in equation (4), results in considerably improved global fit measures such as the DIC and log(psML). The estimated model dimension d_*e *_is actually lower than model 1, possibly indicating a higher degree of pooling strength under this model [15]. There is also improved fit in the lowest age band with the average log(CPO) in the 0–4 age band falling from -9 to -8.3 (Table 2) and the number death counts in 0–4 age group not predicted well falling to 30. On the other hand, deaths at ages over 90 still show discrepant predictions.

**Figure 1:**
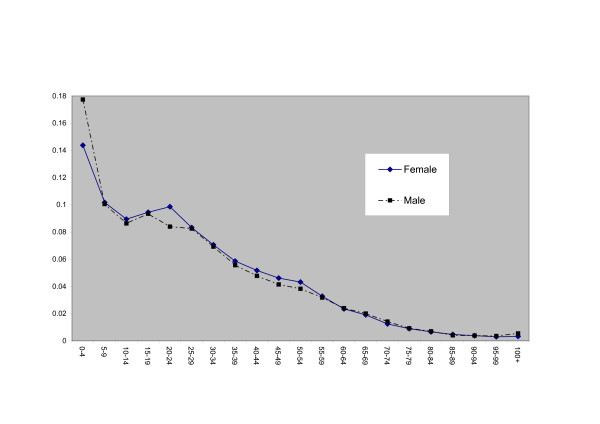
Age Weights, Model 2.

**Figure 2:**
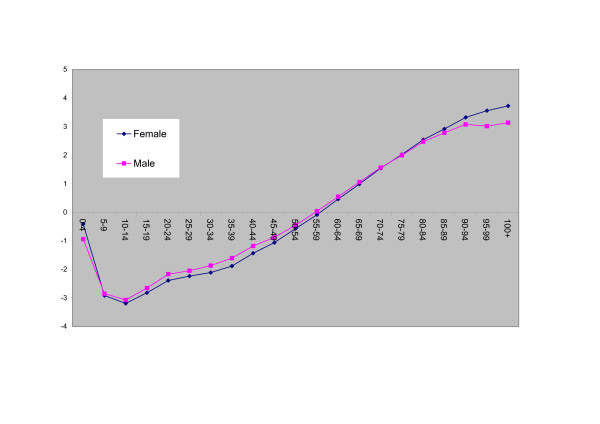
Main Age Effects Model 2.

The division parameters *θ*_*is *_in equation 4 (see Table 3) are highest in the southwestern divisions of Yunnan, Qinghai, Tibet and Guizhou (relatively undeveloped in economic terms, with below average income per head – see Appendix 1), and lowest in developed divisions such as Beijing, Shanghai and Tianjin. Figure 1 plots shows the male and female age weights *φ*_*xs*_; these show that child and young adult mortality is most strongly linked to spatial mortality inequalities. The main age effects *η*_*xs *_(Figure 2) show the expected pattern of increase with age.

**Table 3:** Spatial Effects Model 2

	Female			Male		
	Mean	2.5%	97.5%	Mean	2.5%	97.5%

Beijing	-5.6	-7.0	-5.8	-4.4	-5.6	-2.4
Tianjin	-5.8	-6.8	-6.0	-4.6	-5.5	-3.5
Hebei	-2.7	-3.3	-2.7	-1.9	-2.6	-1.1
Shanxi	-2.1	-2.9	-2.2	-1.3	-2.2	-0.8
Inner Mongolia	-0.2	-0.9	-0.2	0.1	-0.6	0.7
Liaoning	-2.3	-3.3	-2.3	-1.7	-2.3	-1.1
Jilin	-0.7	-1.5	-0.8	-0.5	-1.3	0.8
Heilongjiang	-2.8	-3.5	-2.8	-1.9	-2.5	-0.6
Shanghai	-6.6	-8.1	-7.3	-5.4	-6.7	-2.9
Jiangsu	-3.4	-4.4	-3.5	-2.9	-3.5	-2.0
Zhejiang	-2.3	-2.9	-2.2	-1.7	-2.2	-0.8
Anhui	-0.4	-1.8	-0.3	-0.5	-1.5	0.3
Fujian	-0.9	-1.8	-0.9	-0.5	-1.3	0.4
Jiangxi	1.7	-0.1	2.1	1.2	-0.5	2.3
Shandong	-1.8	-2.6	-1.8	-1.4	-2.2	-0.7
Henan	-1.1	-2.2	-1.1	-0.9	-1.6	-0.2
Hubei	0.3	-0.4	0.3	0.1	-0.5	0.9
Hunan	1.1	0.3	1.0	0.8	0.1	1.4
Guangdong	-1.4	-2.3	-1.4	-1.0	-1.8	0.0
Guangxi	0.5	-1.3	0.6	0.6	-0.6	1.3
Hainan	-1.0	-3.1	-0.9	-1.0	-2.2	-0.2
Chongqing	2.1	0.8	2.2	1.6	-0.1	2.4
Sichuan	2.2	1.5	2.1	1.6	1.0	2.3
Guizhou	6.7	5.9	6.6	5.1	3.4	6.2
Yunnan	6.8	5.9	6.8	5.3	4.5	6.0
Tibet	9.2	8.3	9.2	6.8	5.3	7.6
Shaanxi	1.0	-0.7	1.1	0.8	-0.2	1.6
Gansu	1.8	0.8	1.8	1.3	0.6	1.9
Qinghai	4.7	4.1	4.6	3.5	2.6	4.1
Ningxia	1.0	-0.9	1.1	1.1	0.2	1.8
Xinjiang	2.1	1.2	2.1	1.6	0.9	2.5

In an attempt to reduce model discrepancies further, model 3 is applied with *Pr*(*δ*_*x *_= 1) = *π*_*δ *_= 0.05. This model produces a further improvement in the global fit measures and reduces predictive discrepancies at age group level (Table 2). As can be seen from Table 1, the better fit comes at the cost of a much higher effective parameter count with *d*_*e *_= 346.

One may expect from the discrepancy pattern for model 2, that the need for additional age-area interactions might be greater at the youngest and oldest ages. In fact, there is unequivocal evidence that *ψ*_*xis *_are needed at ages 0–4 and for the last three age bands (90–95, 95–99, 100+) where the posterior probability for inclusion is *Pr*(*δ*_*x *_= 1|*y*) = 1. That is, age-area interactions are retained at these ages in all MCMC iterations. For the 85–90 age band, *Pr*(*δ*_*x *_= 1|*y*) = 0.95. Ten of the remaining probabilities *Pr*(*δ*_*x *_= 1|*y*) are under 0.05, and the remainder range between 0.05 and 0.28. So age-specific area effects are only necessary conclusively for a minority of age groups.

In order to summarise age-sex effects from model 3 in tabular form, the 31 administrative divisions are aggregated to three broad zones [19]: the western zone (the six provinces of Shaanxi, Gansu, Qinghai, Sichuan, Yunnan and Guizhou, the three autonomous regions of Ningxia, Xinjiang and Tibet, and the Chongqing municipality); the eastern and coastal zone (Beijing, Tianjin, Hebei, Liaoning, Shanghai, Jiangsu, Zhejiang, Fujian, Shandong, Guangdong, Guangxi, and Hainan), and the middle zone (Shanxi, Inner Mongolia, Jilin, Heilongjiang, Anhui, Jiangxi, Henan, Hubei, Hunan). Table 4 shows the death rates (posterior means)

**Table 4:** Mortality Rate Profile; Three Zones

	Female			Male		
Age band	East/Coastal	Middle	Western	East/Coastal	Middle	Western

0–4	0.00461	0.00634	0.01124	0.00351	0.00476	0.00810
5–9	0.00040	0.00049	0.00077	0.00059	0.00069	0.00097
10–14	0.00030	0.00036	0.00053	0.00047	0.00054	0.00073
15–19	0.00042	0.00053	0.00080	0.00068	0.00079	0.00108
20–24	0.00060	0.00078	0.00123	0.00110	0.00131	0.00179
25–29	0.00075	0.00092	0.00130	0.00125	0.00146	0.00191
30–34	0.00088	0.00105	0.00139	0.00155	0.00177	0.00220
35–39	0.00119	0.00137	0.00173	0.00202	0.00226	0.00270
40–44	0.00179	0.00204	0.00252	0.00314	0.00347	0.00400
45–49	0.00270	0.00304	0.00363	0.00449	0.00493	0.00559
50–54	0.00457	0.00512	0.00602	0.00686	0.00749	0.00839
55–59	0.00735	0.00804	0.00918	0.01147	0.01231	0.01347
60–64	0.01318	0.01418	0.01564	0.01911	0.02019	0.02146
65–69	0.02269	0.02446	0.02639	0.03165	0.03373	0.03522
70–74	0.03990	0.04241	0.04408	0.05397	0.05666	0.05876
75–79	0.06716	0.07147	0.07339	0.08267	0.08664	0.08842
80–84	0.11190	0.11870	0.12040	0.13480	0.14110	0.14320
85–89	0.16230	0.17140	0.17420	0.18760	0.19500	0.19730
90–94	0.23810	0.25160	0.25350	0.25090	0.25930	0.26330
95–99	0.31650	0.31920	0.31450	0.27430	0.21670	0.21940
100+	0.36970	0.35790	0.36590	0.25320	0.25580	0.26510

**Figure 3:**
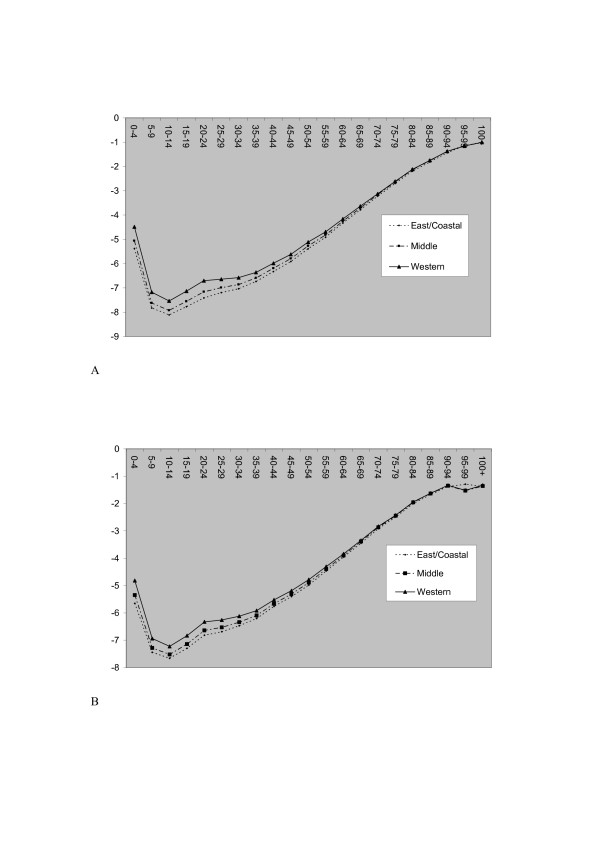
a Female Mortality by Zone. Horizontal Axis Caption: Age group. Vertical Axis Caption: Log rate. b Male Mortality by Zone. Horizontal Axis Caption: Age group. Vertical Axis Caption: Log rate.

*m*_*zsx *_= *ξ*_*zsx*_/*P*_*zsx*_

over the three zones z. Figures 3a and 3b plot the logs of these rates. The latter show the wider contrasts in mortality at lower ages, with excess child and younger adult mortality in the Western zone. Also apparent from Table 4 is higher female than male child mortality (at ages 0–4), as reported elsewhere [20].

## Findings on expectancy variations

Of particular interest as model outputs are the expectancy gap between urban and rural areas, and the contrasts in life expectancy between divisions at different stages of development. As to urban-rural differentials within divisions, the parameter *γ*_*s *_is estimated in model 3 as 0.48 (with standard deviation 0.02) for females, translating into an average rural mortality relative risk of 1.62 with urban areas at 1. For males the corresponding parameter is 0.37 (with relative risk 1.45). So females are particularly disadvantaged by rural location. The rural health disadvantage in modern China has been attributed to the disproportionate subsidies given to urban hospitals, and to the fact that income inequalities between rural and urban areas have been rapidly followed by health inequalities [21]. China's health system has been ranked by the WHO as the lowest in the world in terms of health equity: urban residents make up only about 20% of China's total population, but enjoy about 80% of health resources [22].

Table 5 shows the profile of life expectancies at birth resulting from model 3. These are by administrative division and urban-rural populations within divisions. Also shown are adjusted estimates of life expectancy presented both by Heilig [12] and Cai [9], that were made by China's National Bureau of Statistics [23]. The China wide life expectancies for males and females according to the NBS are respectively 69.6 and 73.3. Cai [9] mentions that "although the NBS did not provide information on how the adjustments were made, the comparison indicates that adjustments were made for the undercounting of deaths in the census". The China wide life expectancies for males and females from model 3 are respectively 69.7 and 72.7. These are very close to estimates made by Banister & Hill [8], that also adjust for death undercounting, namely 69.7 and 72.8.

Table 5 contains estimates (namely posterior means) obtained by sampling at each MCMC iteration, but in fact one advantage of the Bayesian sampling approach is that the full density of expectancies and other life table indices can be obtained. Most density plots for the 124 expectancies at birth are symmetric (i.e. do not show skewness inconsistent with normality) with discrepancies between mean and median under 0.1 year. However, many posterior plots of life expectancy at birth exhibit levels of kurtosis inconsistent with posterior normality. For examples, analysis of samples of size 1000 from the 62 posterior urban life expectancy densities found 12 with significant positive kurtosis (and none with significant negative kurtosis). So assessing whether life expectancy at birth E_0*i *_in division i exceeded that in division j might be problematic under classical analysis, but under a Bayesian sampling approach the posterior probability *Pr*(*E*_0*i *_> *E*_0*j*_*|y*) is easily obtained.

Table 5 shows generally higher expectancies for women; men have considerably higher tobacco and alcohol consumption than women in China [24,25], and are involved in more traffic accidents and work-related diseases and deaths than women [26]. However, women show a greater rural vs urban survival disadvantage than men, and their excess life expectancy over males is smaller in rural areas (71 vs 68.3 for males), whereas in urban areas the excess averages 3.8 years (77.1 vs 73.3).

**Figure 4:**
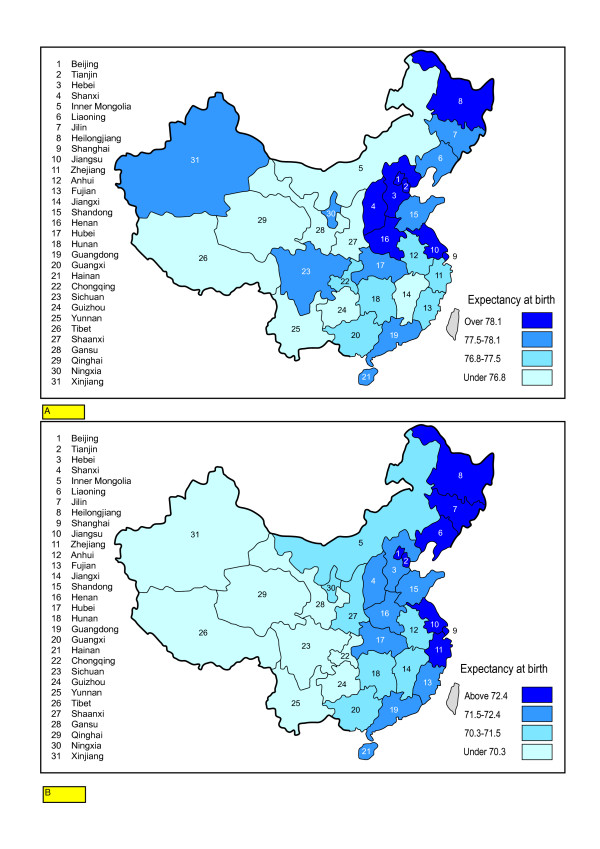
a Female Expectancy Urban Subdivisions. b Female Expectancy, Rural Subdivisions.

There are wider expectancy contrasts in both female and male expectancies within the rural sub-divisions than the urban sub-divisions of the 31 regions. For females, the range in urban subdivisions is from 72.4 (Yunnan) to 80.1 (Shanghai), while for rural subdivisions, the range is from 64.5 (Yunnan) to 75.2 (Shanghai). Figures 4a and 4b show female expectancies for urban and rural female populations in each division; they correlate highly, showing that rural populations within healthier divisions benefit in improved survival terms as well as urban population groups.

## Conclusion

This paper has sought to show the gains of using a model based approach to life tables for a set of regions. It has considered mortality in 31 Chinese administrative divisions and shown how the major aspects of mortality variation can be summarised in parsimonious models with interpretable parameters. In terms of geographic contrasts the main results are the wide inter-division and urban-rural contrasts in mortality risks and life expectancy.

The modelling approach allows the significance of such contrasts to be readily assessed – and indeed the full posterior sampling density of life table measures such as life expectancy can be obtained by using MCMC sampling output. By contrast, classical analysis relies on approximations such as Chiang's "delta method" approximation [27] of the sample variances of life expectancies; such approximations have to be worked out for each life table measure separately.

The model schemes used have shown that area and age effects do not conform to the multiplicative model considered by Hoem [5] and others – the multiplicative model is equivalent to assuming effects of age and area are additive in the log mortality risk scale. Instead, it is found that spatial contrasts in Chinese division mortality are especially apparent in younger age groups and model fit (for models 2 and 3) is greatly improved by allowing for such non-proportionality.

Much of the disease mapping literature, especially that adopting a Bayesian modelling approach, has assumed additive effects in the log relative risk scale, or has eliminated consideration of age (or other demographic stratifiers) using indirect standardisation, often without prior checks on the validity of such simplifications. The indirect standardisation approach [6] applies a standard age schedule (e.g. national age mortality rates) across all areas and leads to models where event counts y_*i *_for area i are compared to expected deaths E_*i *_which are treated as an offset.

The present research emphasizes the importance of evaluating such basic assumptions in making epidemiological or health inferences, since relative risk contrasts between areas may not be the same when age-area interactions are allowed for. If mortality and health inequalities between divisions or urban-rural areas are strongly age related then this may be important in strategic health interventions to counteract health imbalances.

While having implications for health policy, the type of analysis undertaken in this paper is descriptive in the sense that one is not seeking to explain life expectancy contrasts in terms of a range of potential risk factors: health behaviours, environmental or climatic factors, income levels or income inequality, ethnicity and/or nationality, education and literacy levels, female status, or economic structure [28,29]. Rather a statistical modelling approach accounting for age and area effects can be seen as a preliminary to, but also providing a distinct perspective to, a causal model that would typically just examine total life expectancy as a response.

## Appendix 1 socioeconomic characteristics of administrative divisions

Table 6 shows selected socio-economic features of the administrative divisions. The indicators are based on the 2000 census (based on tabulations distributed by China Data Center at the University of Michigan), except for data on GDP per head from the work of Heilig [1]. To summarise ethnicity/nationality, the percent Han Chinese is used: of the total population of 1198 million people living in the 31 provinces, autonomous regions and municipalities, 91.6% were of Han nationality. At division level the variation is from 6 to 100%.

**Table 5:** Life Expectancies at Birth, by Administrative Division & Urban-Rural Populations within Divisions Model 3 Smoothed Expectancies (Urban, Rural, All Populations) vs Official Life Tables (All Populations)

	FEMALES				MALES			
	MODEL			OFFICIAL	MODEL			OFFICIAL

Admin Division	Urban	Rural	All	All	Urban	Rural	All	All

Beijing	79.6	74.6	78.3	78.0	75.4	71.2	74.3	74.3
Tianjin	79.7	74.6	77.9	76.6	75.6	71.3	74.0	73.3
Hebei	78.2	72.5	74.0	74.6	74.2	69.7	70.8	70.7
Shanxi	78.1	72.4	74.2	73.6	74.1	69.4	70.9	70.0
Inner Mongolia	76.9	70.8	73.4	71.8	73.2	68.1	70.3	68.3
Liaoning	78.3	72.8	75.5	75.4	74.2	69.6	71.8	71.5
Jilin	77.9	72.0	74.8	75.0	73.9	69.1	71.4	71.4
Heilongjiang	78.8	73.3	76.0	74.7	74.7	70.0	72.3	70.4
Shanghai	80.1	75.2	79.5	80.0	76.2	72.1	75.7	76.2
Jiangsu	78.7	73.2	75.6	76.2	74.8	70.2	72.1	71.7
Zhejiang	78.4	72.9	75.4	77.2	74.3	69.8	71.8	72.5
Anhui	77.2	71.0	72.6	73.6	73.6	68.7	69.9	70.2
Fujian	77.6	71.7	74.0	75.1	73.8	69.1	70.9	70.3
Jiangxi	75.4	68.5	70.3	69.3	72.3	67.2	68.4	68.4
Shandong	77.8	72.1	74.3	76.3	73.9	69.2	71.0	71.7
Henan	77.6	71.5	72.9	73.4	73.8	69.0	70.0	69.7
Hubei	77.7	71.7	74.2	73.0	73.7	68.9	70.8	69.3
Hunan	77.0	70.8	72.5	72.5	73.1	68.0	69.3	69.1
Guangdong	77.9	72.2	74.8	75.9	74.0	69.4	71.6	70.8
Guangxi	76.9	70.4	71.9	73.8	73.3	68.3	69.5	69.1
Hainan	77.8	71.7	73.9	75.3	74.3	69.5	71.4	70.7
Chongqing	76.8	70.6	72.3	73.9	72.7	67.5	68.8	69.8
Sichuan	76.9	70.6	72.1	73.4	72.6	67.5	68.6	69.3
Guizhou	72.9	65.1	66.6	67.6	69.2	63.2	64.4	64.5
Yunnan	72.4	64.5	66.1	66.9	69.2	63.3	64.4	64.2
Tibet	73.0	65.3	66.3	66.2	68.9	62.8	63.6	62.5
Shaanxi	76.3	69.8	71.8	71.3	73.3	68.3	69.7	68.9
Gansu	75.9	69.1	70.5	68.3	72.7	67.2	68.3	66.8
Qinghai	74.9	68.0	69.7	67.7	71.1	65.4	66.9	64.6
Ningxia	77.4	71.3	72.8	71.8	72.9	67.8	69.1	68.7
Xinjiang	76.6	69.8	71.7	69.1	72.2	66.8	68.4	66.0
China	77.1	71.0	72.7	73.3	73.3	68.3	69.7	69.6

**Table 6:** Socioeconomic Characteristics of Administrative Divisions*

Division	Illiteracy (% total popn over 15)	Female Illiteracy (% Fem Popn 15+)	% Popn classified as rural	GDP per head (2003) in $	% of Non-agricutural Population	% Han
Total	9.1	13.5	63	4726	25	92
Beijing	4.9	8.1	22	16649	60	96
Tianjin	6.5	10.2	28	13778	55	97
Hebei	8.6	10.8	74	5459	19	96
Shanxi	5.7	8.3	65	3861	26	100
Inner Mongolia	11.6	16.5	57	4660	35	79
Liaoning	5.8	8.7	45	7404	46	84
Jilin	5.7	8.1	50	4849	43	91
Heilongjiang	6.3	9.1	48	6032	47	95
Shanghai	6.2	10.3	12	24260	63	99
Jiangsu	7.9	12.3	58	8729	29	100
Zhejiang	8.6	12.9	51	10462	21	99
Anhui	13.4	19.5	73	3352	18	99
Fujian	9.7	14.0	58	7778	20	98
Jiangxi	7.0	11.0	72	3468	23	100
Shandong	10.8	16.0	62	7094	21	99
Henan	7.9	11.7	77	3931	17	99
Hubei	9.3	14.5	60	4679	27	96
Hunan	6.0	9.5	73	3923	20	90
Guangdong	5.2	8.6	44	8938	26	99
Guangxi	5.3	8.9	72	3100	18	62
Hainan	9.7	16.1	59	4318	31	83
Chongqing	8.9	13.5	67	3744	22	94
Sichuan	9.9	14.6	73	3333	18	95
Guizhou	19.9	30.6	76	1871	15	62
Yunnan	15.4	22.2	77	2940	15	67
Tibet	47.3	60.5	81	3568	13	6
Shaanxi	9.8	14.2	68	3365	22	100
Gansu	19.7	27.8	76	2608	19	91
Qinghai	25.4	35.9	68	3779	27	54
Ningxia	15.7	22.3	68	3475	28	65
Xinjiang	7.7	9.9	66	5037	30	41

* Source: Census 2000, except for income data which is from Heilig 2006 (Appendix 1)

## Appendix 2 methods: details on priors for models

A fully Bayesian strategy is adopted using Monte Carlo Markov Chain (MCMC) estimation. For bivariate spatial effects, namely *λ*_*i *_= (*λ*_*i*1_, *λ*_*i*2_) in model 1 and *θ*_*i *_= (*θ*_*i*1_, *θ*_*i*2_) in models 2 & 3, a bivariate version of the CAR normal prior [30] is assumed. Thus with n = 31 provinces, and with i and j denoting different provinces, the pairwise difference prior for *λ*_*i *_= (*λ*_*i*1_, *λ*_*i*2_) has precision matrix Ψ_*λ *_and joint density

P(λi|Ψλ)∝|Ψλ|n/2exp⁡{−∑i,jwij(λi−λj)′Ψλ(λi−λj)}
 MathType@MTEF@5@5@+=feaafiart1ev1aaatCvAUfKttLearuWrP9MDH5MBPbIqV92AaeXatLxBI9gBaebbnrfifHhDYfgasaacH8akY=wiFfYdH8Gipec8Eeeu0xXdbba9frFj0=OqFfea0dXdd9vqai=hGuQ8kuc9pgc9s8qqaq=dirpe0xb9q8qiLsFr0=vr0=vr0dc8meaabaqaciaacaGaaeqabaqabeGadaaakeaacqWGqbaucqGGOaakiiGacqWF7oaBdaWgaaWcbaGaemyAaKgabeaakiabcYha8jabfI6aznaaBaaaleaacqWF7oaBaeqaaOGaeiykaKIaeyyhIuRaeiiFaWNaeuiQdK1aaSbaaSqaaiab=T7aSbqabaGccqGG8baFdaahaaWcbeqaaiabd6gaUjabc+caViabikdaYaaakiGbcwgaLjabcIha4jabcchaWjabcUha7jabgkHiTmaaqafabaGaem4DaC3aaSbaaSqaaiabdMgaPjabdQgaQbqabaGccqGGOaakcqWF7oaBdaWgaaWcbaGaemyAaKgabeaakiabgkHiTiab=T7aSnaaBaaaleaacqWGQbGAaeqaaOGafiykaKIbauaaaSqaaiabdMgaPjabcYcaSiabdQgaQbqab0GaeyyeIuoakiabfI6aznaaBaaaleaacqWF7oaBaeqaaOGaeiikaGIae83UdW2aaSbaaSqaaiabdMgaPbqabaGccqGHsislcqWF7oaBdaWgaaWcbaGaemOAaOgabeaakiabcMcaPiabc2ha9baa@6B3C@

A contiguity assumption is made for the geographic interactions so that *w*_*ij *_= 1 for adjacent provinces and zero otherwise. A Wishart prior with identity scale matrix and 2 degrees of freedom is adopted on Ψ_*λ *_. The spatial effects are centred at each MCMC iteration (to sum to zero over all provinces).

The age effects *η*_*xs *_in all three models are modelled in terms of normal autoregressive random walks with variances *ω*_*s *_specific to sex, so

*η*_*xs *_~ *N*(*η*_*x*-1,*s*_, *ω*_*s*_)

This prior does not set a level and so male and female age effects are centred at each MCMC iteration (using the car.normal prior in WINBUGS).

A uniform U(0,1000) prior is assumed for the negative binomial *α*, and N(0,1000) priors on the fixed effects {*k*_*s*_, *γ*_*s*_}. For the precisions *ω*_*s *_(in all models) and *ζ*_*x *_(in model 3), a uniform U(0,1000) prior is assumed. The age weight parameters *φ*_*xs *_in models 2 and 3 are generated using gamma Ga(l,l) priors on parameters *f*_*xs*_, and then scaling the *f*_*xs *_to sum to 1 within genders s. So

ϕxs=fxs/∑xfxs
 MathType@MTEF@5@5@+=feaafiart1ev1aaatCvAUfKttLearuWrP9MDH5MBPbIqV92AaeXatLxBI9gBaebbnrfifHhDYfgasaacH8akY=wiFfYdH8Gipec8Eeeu0xXdbba9frFj0=OqFfea0dXdd9vqai=hGuQ8kuc9pgc9s8qqaq=dirpe0xb9q8qiLsFr0=vr0=vr0dc8meaabaqaciaacaGaaeqabaqabeGadaaakeaaiiGacqWFvpGAdaWgaaWcbaGaemiEaGNaem4Camhabeaakiabg2da9iabdAgaMnaaBaaaleaacqWG4baEcqWGZbWCaeqaaOGaei4la8YaaabuaeaacqWGMbGzdaWgaaWcbaGaemiEaGNaem4CamhabeaaaeaacqWG4baEaeqaniabggHiLdaaaa@3FF6@

This is equivalent to a Dirichlet prior on the *φ*_*xs*_^17^.

## Appendix 3 methods: model assessment

Comparisons of model fit in the analysis use two criteria: the deviance information criterion (DIC) of Spiegelhalter et al [31], and the pseudo marginal likelihood (psML) based on Monte Carlo estimates of conditional predictive ordinates, or CPOs [32,33]. The DIC is obtained as the posterior mean deviance plus a measure of complexity d_*e *_that can also be regarded as estimating the effective dimension of the model, but also reflects features such as parameter precision.

The CPO is a cross-validatory measure of predictive fit, namely

*CPO *= *p*(*y*_*h*_|*y*_[*h*]_, *θ*)

where *θ *are the model parameters, y_*h *_indicates deaths in a particular one of the 2604 subdivision-area-gender-age strata, and y_[*h*] _denotes the data in the remaining 2603 strata. The conditional predictive ordinates are indicators of model adequacy at the level of individual observations; low CPOs (or highly negative log CPOs) indicate poorly fitted points. The overall total of log(CPO) provides a log(psML) which will be higher for a model providing a better overall fit.

Model adequacy may also be assessed using predictions from the model – more specifically, replicate observations y_*rep*,*risx *_sampled from the posterior predictive density p(y_*rep*_|y). Following Gelfand [34], a summary of how well these predictions match the actual data, y_*risx*_, involves a tally of how many actual observations are located within the 95% interval of the corresponding model prediction y_*rep*,*risx*_. If 95% or more of the *N *= 2 × 31 × 2 × 21 = 2604 death counts y_*risx *_are within the 95% intervals of the predictions then the model is judged to be reproducing the observations satisfactorily.

## References

[B1] Heilig A (2006). Many Chinas?: the economic diversity of China's provinces. Population & Development Review.

[B2] Liu G, Wu X, Peng C, Fu A (2003). Urbanization and health care in rural China. Contemporary Economic Policy.

[B3] Population Analysis Spreadsheets. http://www.census.gov/ipc/www/pas.html.

[B4] Zhu L, Gorman D, Horel S Hierarchical Bayesian spatial models for alcohol availability, drug "hot spots" and violent crime. Int J Health Geogr.

[B5] Hoem J (1987). Statistical analysis of a multiplicative model and its application to the standardization of vital rates: a review. International Statistical Review.

[B6] Clayton D, Kaldor J (1987). Empirical Bayes estimates of age-standardized relative risks for use in disease mapping. Biometrics.

[B7] Lee R, Carter D (1992). Modeling and forecasting the time series of U.S. mortality. J Amer Statist Assoc.

[B8] Banister J, Hill K (2004). Mortality in China 1964–2000. Popul Stud.

[B9] Cai Y (2005). National, provincial, prefectural and county life tables for China based on the 2000 Census. CSDE Working Paper 05-03.

[B10] Library of Congress, Federal Research Division (2006). Country Profile: China.

[B11] Hill K (1987). Estimating census and death registration completeness. Asian and Pacific Population Forum.

[B12] Heilig A (2007). The China-Profile: Facts, Figures, and Analyses.

[B13] Lai D (2005). Decomposition of Chinese life expectancy via cccupation. Population and Environment.

[B14] Lai D, Guo F, Hardy R (2000). Standardized mortality ratio and life expectancy: a comparative study of Chinese mortality. International Journal of Epidemiology.

[B15] Carlin B, Clark J, Gelfand A, Clark J, Gelfand A (2006). Elements of hierarchical Bayesian inference. Hierarchical Modelling for the Environmental Sciences: Statistical Methods and Applications.

[B16] Best N, Lawson A, Biggeri A, Boehning D, Lessafre E, Viel J, Bertollini R (1999). Bayesian Ecological Modelling. Disease Mapping and Risk Assesment for Public Health.

[B17] Lunn D, Thomas A, Best N, Spiegelhalter D (2000). WinBUGS A Bayesian modelling framework: concepts, structure, and extensibility. Statistics and Computing.

[B18] Gelman A, Carlin J, Stern H, Rubin D (2004). Bayesian Data Analysis.

[B19] Zha B (1996). The gap of economic development expanding between eastern China and middle, western China. China Popul Res Newsl.

[B20] Xu B, Rimpela A, Jarvelin M, Nieminen M (1994). Sex differences of infant and child mortality in China. Scand J Soc Med.

[B21] The Lancet Editorial (2004). China must prioritize health opportunities for all. The Lancet.

[B22] Li C (2001). Health care in rural China: current development and strategic planning. Chinese Health Econ.

[B23] National Bureau of Statistics (2003). China Statistical Yearbook.

[B24] Cochrane J, Chen H, Conigrave K, Hao W (2003). Alcohol use in China. Alcohol & Alcoholism.

[B25] Yang G, Fan L, Tan J, Qi G, Zhang Y, Samet J, Taylor C, Becker K, Xu J (1999). Smoking in China: Findings of the 1996 National Prevalence Survey. JAMA.

[B26] International Labour Organisation (2002). Global Estimates of Fatalities Caused by Work Related Diseases and Occupational Accidents.

[B27] Chiang C (1984). The Life Table and Its Applications.

[B28] Hricko A (1994). Environmental problems behind the Great Wall. Environ Health Perspect.

[B29] Zhao Z (2006). Income inequality, unequal health care access, and mortality in China. Population and Development Review.

[B30] Gamerman D, Moreira A, Rue H (2003). Space-varying regression models: specifications and simulation. Computational Statistics & Data Analysis.

[B31] Spiegelhalter D, Best N, Carlin B, van der Linde A (2002). Bayesian measures of model complexity and fit. Journal of the Royal Statistical Society, Series B.

[B32] Silva R, Lopes H, Migon H (2006). The extended generalized inverse Gaussian distribution for log-linear and stochastic volatility models. Brazilian Journal of Probability & Statistics.

[B33] Sinha D, Dey D (1997). Semiparametric Bayesian analysis of survival data. J Amer Statist Assoc.

[B34] Gelfand A, Gilks W, Richardson S, Spieglehalter D (1996). Model determination using sampling based methods. Markov Chain Monte Carlo in Practice.

